# Facial Complexity in Sun Bears: Exact Facial Mimicry and Social Sensitivity

**DOI:** 10.1038/s41598-019-39932-6

**Published:** 2019-03-21

**Authors:** Derry Taylor, Daniela Hartmann, Guillaume Dezecache, Siew Te Wong, Marina Davila-Ross

**Affiliations:** 1 Psychology Department, University of Portsmouth, King Henry Building, Portsmouth, PO1 2DY United Kingdom; 2 Justus Liebig University Gießen, Department of Special Needs and Clinical Child and Adolescent Psychology. Otto-Behaghelstraße 10E, D35394 Gießen, Germany; 3 Institut Jean Nicod, Département d’études cognitives, ENS, EHESS, CNRS, PSL Research University, Paris, France; 4 Bornean Sun Bear Conservation Centre, PPM 219, Elopura, Sandakan, 90000 Sabah Malaysia

## Abstract

Facial mimicry is a central feature of human social interactions. Although it has been evidenced in other mammals, no study has yet shown that this phenomenon can reach the level of precision seem in humans and gorillas. Here, we studied the facial complexity of group-housed sun bears, a typically solitary species, with special focus on testing for exact facial mimicry. Our results provided evidence that the bears have the ability to mimic the expressions of their conspecifics and that they do so by matching the exact facial variants they interact with. In addition, the data showed the bears produced the open-mouth faces predominantly when they received the recipient’s attention, suggesting a degree of social sensitivity. Our finding questions the relationship between communicative complexity and social complexity, and suggests the possibility that the capacity for complex facial communication is phylogenetically more widespread than previously thought.

## Introduction

Behavioural mimicry pervades human social interactions^1^. Facial, postural and vocal signals are regularly automatically shared by others, sometimes resulting in or from emotional contagion^2^. There has been a vast literature on facial mimicry^3^, leading to the view that humans are capable of matching the facial expressions of others with great precision^4^ (hereafter, “exact facial mimicry”), with benefits during social interactions such as strengthened social bonds and sharing detailed emotional information^2,5^. Although it has been shown that facial mimicry is also present in non-human mammals^6–8^, only one study of gorillas (*Gorilla gorilla gorilla*)^9^ has so far demonstrated the precision as seen in humans, i.e., by mimicking one variant of a facial display over another.

In this study, we examined facial expressions in spontaneous social play of group-housed sun bears (*Helarctos malayanus*). Many basic facts of sun bear biology are unknown, due to the difficulties of studying this elusive species in the wild^10,11^. Nonetheless, it is known that sun bears feed on an omnivorous diet in tropical rainforests^12^, and a study on adult sun bears in Ulu Segama Forest Reserve showed that they seldom participate in social interactions with one another outside of mating contexts despite home ranges overlapping by up to 20%^10^, indicating a largely solitary lifestyle. Notably, mothers often raise two offspring simultaneously, which are highly altricial for the first 3 months and interact with the mother extensively during this period^11^. Facial expressions in sun bears have not been studied, but open-mouth expressions are shown mostly by juveniles and during play in the closely related American black bears (*Ursus americanus*)^13^.

Sun bears use two distinct variants of open-mouth faces during play (personal observations), similar to American black bears^13^ and other carnivorans^14^. This observation is intriguing because it raises the possibility that sun bears exhibit complex forms of facial communication comparable to those that have been shown mostly in species with strong social tendencies^6–9^. In turn, this implies that complex forms of communication cannot be explained only as evolved adaptations to a demanding social environment^15^.

Hallmarks of complex facial communication include for instance muscular variation in expressions^15^, facial mimicry^1,3,4^, and social sensitivity to the attentional states of others during expression production^16–18^. As facial mimicry occurs in phylogenetically distanced mammalian species during play (primates^6,7,9^; dogs^8^) and sun bears produce distinct facial variants during social play which is an essential precondition for exact mimicry, our hypothesis is that facial mimicry and exact facial mimicry are present in sun bears during social play. Additionally, social sensitivity was also measured as a component of such complexity. Social sensitivity via facial communication was previously suggested for dogs (*Canis familiaris*)^16^ and apes^17,18^, mammalian taxa that are closely associated with humans on a social and phylogenetic level, respectively. Social sensitivity is essential to effective facial communication, particularly during play wherein the absence of play signalling often escalates play into aggression^19^. Given that bears are known to engage in play^13^, we therefore expect sun bears show social sensitivity in their facial expression production.

## Material and Method

### Sun bears and data collection

Twenty-two group-housed rehabilitant sun bears (aged 2–12 years; mean age = 6.0 ± 2.9 SD) of the Bornean Sun Bear Conservation Centre (Malaysia) were studied. All bears were unrelated. The bears were video-recorded using 3-minute focal recordings and ad libitum recordings from January 2015 to September 2016 and from August to December 2017. Recordings of the bears were collected in three outdoor forest enclosures, ranging from 0.13–0.32 hectares, meaning enclosures were large enough that bears did not have to socially interact by necessity. Group compositions were changed throughout the data collection of this study, but the group sizes within enclosures did not exceed six bears. For further details about these bears (see Table S1), study site and recording equipment, see Supplementary Methods.

### Behavioural coding

Social play involved one bear directing a play action towards another bear, and the other bear responding with a play action. Social play began with the first play action and ended when play actions stopped for 10 seconds or more. Three-hundred seventy-two social play bouts were identified. Within these play bouts, scenes of rough play were observed 135 times and scenes of gentle play were observed 333 times (see supplementary electronic materials for examples and definitions of rough and gentle play). A single play bout could include both rough and gentle play at different points. Nine-hundred and thirty-one open-mouth facial expressions were coded during these bouts when the mouth opened widely and the jaw dropped. All observed expressions were coded. Two variants were identified: WUI (With Upper Incisors) occurred when the upper lip and nose were raised, which resulted in the wrinkling of the muzzle bridge and the revealing of the upper incisors (i.e., the row of small teeth between the two canine teeth) (n = 450); NUI (No Upper Incisors) occurred when the bear did not raise the upper lip and nose to such an extent, displaying therefore no wrinkling of the muzzle bridge and no upper incisors (n = 481 expressions). During social play, it was coded whether playmates were facing each other. Bears had to be within a 45-degree head rotation to be considered face-to-face. Inter-coder reliability tests showed Kappa values of 0.73 for facial variants as well as 0.80 for facing (both based on 102 expressions – 10.9% of the sample) and 0.75 for play intensity (based on 51 play bouts – 13.7% of the sample). For additional details on the behavioural coding, see Supplementary Methods Tables S2–S4.

### Data analyses

Facial mimicry is a phenomenon whereby a facial expression of a subject is triggered specifically by a similar facial expression it has just observed in another individual (See supplementary electronic materials). Here, the subject was always the individual who perceived an expression, the individual who first produced an expression was always the ‘playmate’. To examine whether subjects showed facial mimicry, we used the method developed by Davila-Ross and colleagues^6,20^. We first coded whether a subject produced an open-mouth face within one second of perceiving an open-mouth face in their playmate while face-to-face; such types of scenes were named ‘scene 1’. We then searched for comparable scenes, where the same dyad was engaging in the same play intensity while face-to-face, but wherein the playmate was not showing an open-mouth face; such scenes were named ‘scene 2’. We then coded whether the subject showed an open-mouth face within 1 second of the onset of scene 2. Combining the two types of scenes allows assessment of whether subjects’ facial behaviour is influenced by playmates’ facial behaviour while controlling for other relevant variables, i.e., dyad composition and play intensity^20^. The starting point for locating a scene 2 was 5 seconds following a scene 1. If the playmate was producing an open-mouth face at this point, the search was continued linearly until a scene wherein the playmate was not producing an open-mouth face was found. Together, scene 1 and 2 gave rise to 4 possible case types: subject shows an open-mouth face only in scene 1, subject only shows an open-mouth face in scene 2, subject always shows an open-mouth face, and subject never shows an open-mouth face. If sun bears show facial mimicry, they should be significantly more likely to produce open-mouth faces in the first case type compared to the second case type. The comparison of these case types test directly if the open-mouth faces of the subjects are actual responses to open-mouth faces of the playmates and, thus, this method represents a highly controlled quantitative manner to gauge responses to a specific stimulus in natural social settings^6^.

**Figure 1:**
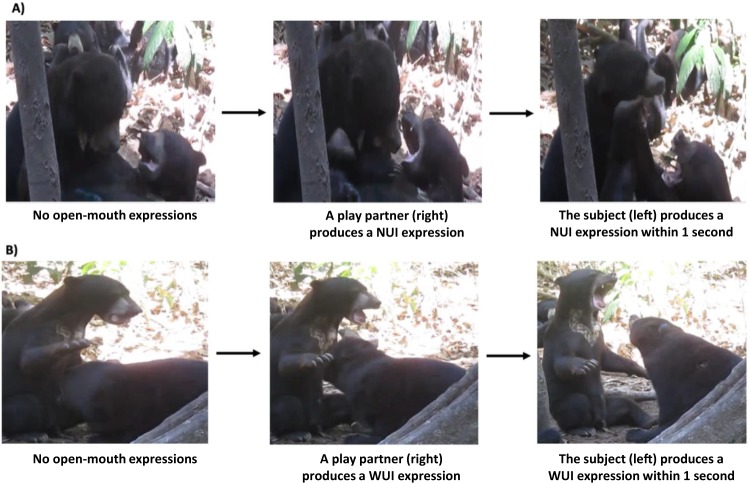
Exact matching of open-mouth variants. Series of photographs demonstrating exact matching of (**A**) NUI open-mouth expression and (**B**) WUI open-mouth expression.

Afterwards, we examined whether the subjects responded to their playmates’ open-mouth faces with exactly matching expressions. Specifically, we coded exactly matching when a given subject displayed a facial variant that matched the facial variant produced by their playmate emitted within 1 second prior to it (e.g., NUI following NUI). We coded ‘Non-exact’ behaviour otherwise (e.g., NUI following WUI). See Fig. 1 for an example. The number of times each subject matched the perceived variant was compared to the number of times each subject produced a non-exact variant. If a significantly greater number of expressions were exactly matching rather than non-exact, then it would represent evidence of exact facial mimicry in sun bears. Only expressions produced when face-to-face were included in all mimicry analyses because an expression not seen could by definition not be mimicked.

To examine social sensitivity, we compared the total number of expressions per subject that were produced when face-to-face versus not face-to-face. Finally, to explore the role of open-mouth expressions and facial matching during social play, rates of expression production and rapid responses (both exactly matching and non-matching) were calculated by dividing the number of expressions produced or the total number of instances of rapid facial responses by the amount of time spent engaging in rough or gentle social play. These rates were then compared within and between play intensities. The relationship between rates of expression production, mimicry, and play duration was also examined. For further analyses (on play intensity), see Supplementary Methods. For multiple comparisons, Bonferroni corrections were used.

## Results

Of the 22 bears studied, 21 produced open-mouth expressions, and 13 showed them within 1 second following the open-mouth face of a playmate while face-to-face. A mean of 19.5% (±21.7% SD) of open-mouth expressions produced by the playmates when facing each other was followed by an open-mouth expression within 1 second. Percentages per subject can be seen in Supplementary Results Table S5.

### Testing for facial mimicry

**Figure 2:**
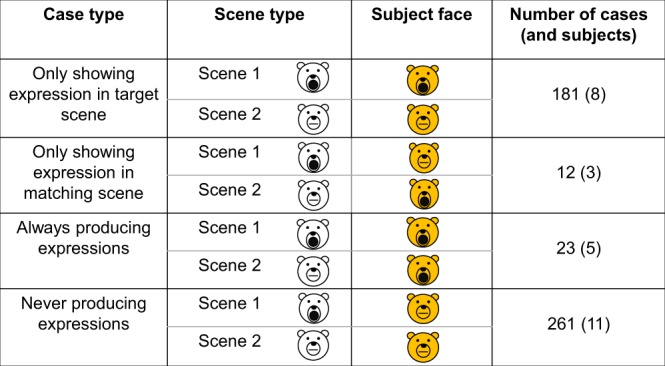
Number of each case type observed in total. The white face on the left represents the playmate, and the orange face on the right represents the subjects’ facial behaviour within the following second. Circular mouths correspond to a facial expression whereas flat mouths do not.

To test for facial mimicry, we examined whether subjects were more likely to produce their open-mouth expressions as a response to the open-mouth expression in a playmate than as a response to an expression with a closed mouth in a playmate. Subjects showed significantly more expressions in the former (one-tailed McNemar test, χ^2^ = 294.22, p < 0.001) (Fig. 2). See Supplementary Results Table S6 for a breakdown of case types per subject.

### Testing for exact facial mimicry

**Figure 3:**
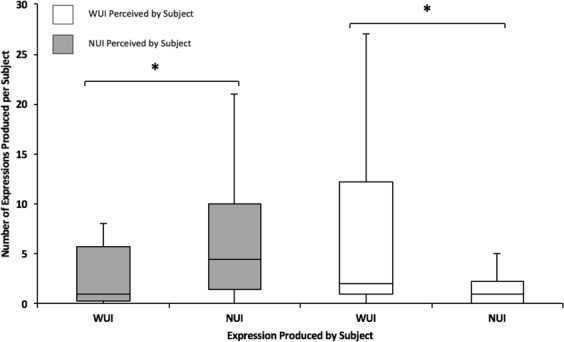
Subject NUI (N = 10 bears) and WUI (N = 12) expressions following NUI and WUI expressions of the social partners within 1 second. The box plots depict medians, upper and lower quartiles, and minimum and maximum range values. *p < 0.05.

Following playmates’ NUI expressions, subjects produced significantly higher numbers of NUI expressions than WUI expressions within 1 second (one-tailed Wilcoxon signed-ranks, Z = −2.61, T = 2, N = 10, p = 0.005); 72.2% (±7.1% SD) of these facial expressions produced by subjects were NUI expressions. Similarly, the subjects showed significantly more WUI expressions than NUI expressions following the playmates’ WUI expressions produced within 1 second prior (Z = −2.30, T = 10, N = 12, p = 0.011) (Fig. 3); 82.2% (±9.0% SD) of these facial expressions produced by subjects were WUI expressions. See Tables S7 and S8 of the Supplementary Results for a breakdown per subject.

### Facial behavior in relation to social sensitivity, play intensity, and play duration

#### Social sensitivity

The bears showed significantly higher occurrences of open-mouth expressions when face-to-face than when not face-to-face (two-tailed Wilcoxon Signed-Ranks, Z = −3.17, T = 1, N = 21 p < 0.001). The bears were facing each other only 14.6% (±5.5% SD) of the play duration.

#### Play intensity

Within gentle play, exact matching of open-mouth faces within 1 second occurred significantly more frequently than matching of open-mouth faces that was not exact (two-tailed Wilcoxon Signed-Ranks, Z = −0.268, T = 1, N = 11, p = 0.042). Within rough play, no such differences were found (Z = −0.943, T = 6, N = 7, p = 0.345). Furthermore, exact matching of NUI variants did not differ significantly from exact matching of WUI variants during gentle play (Z = −0.135, N = 6, p = 0.892), nor during rough play (Z = −0.406, N = 6, p = 0.684). No significant differences between gentle versus rough play were found for overall rates of exact open-mouth matching (Z = −0.710, T = 30, N = 12, p = 0.477), rates of exact NUI matching (Z = −0.1.183, T = 7, N = 7, p = 0.237) and rates of exact WUI matching (Z = −0.700, T = 13, N = 8, p = 0.484). For additional results on play intensity, see Supplementary Results.

#### Play duration

No significant correlation was observed between the rate of expressions produced (total number of expressions divided by the total amount of time spent playing during observations) and the play duration per subject (Spearman’s rank correlation: r_s_(22) = −0.07, p = 0.750). No statistically significant relationship was found between the play bout duration and the rate of facial mimicry; this was the case when all matched expressions (both exact and non-exact) were examined (Spearman’s rank correlation, r_s_(14) = −0.01, p = 0.960) and when only exactly matched open-mouth expressions were examined (Spearman’s rank correlation: r_s_(11) = −0.21, p = 0.500).

## Discussion

This study examined facial communication in sun bears. The results showed bears produced the majority of their open-mouth expressions when their playmates faced them. To our knowledge, this is the first demonstration that the production of facial expressions is sensitive to social partner’s attentional state in a bear species. Thus, even a non-domesticated non-primate mammal is likely to have social sensitivity as part of their communication, comparable to dogs^16^ and apes^17,18^, who modify their facial morphologies when seen by social partners. Such sensitivity may be important for efficiently communicating facial displays. Although, we failed to find any relationship between facial expression production and play duration, in contrast to previous studies^21,22^, indicating that unveiling the function of facial expression production in sun bears requires further investigation.

Special focus of this study was facial mimicry and exact facial mimicry. Firstly, the results suggested that the sun bears mimicked facial expressions of their playmates. Facial expressions of sun bears are, thus, likely to promote communicative exchanges via mimicking similar to dogs^8^, and primates^6,7,9^. Secondly, the results showed that the bears matched the same facial variant of their social partners, suggesting facial mimicry in sun bears to be ‘exact’. This precision in facial replication, shown so far only in humans^4^ and gorillas^9^, was found despite primates arguably having more specialized brain regions for facial processing than other mammals^23^. As neither facial variant was more likely to occur in either rough or gentle play, such exact matching cannot be attributed to particular variants only occurring in particular contexts. Moreover, play vocalizations in the studied sun bears are rare and they were not heard to be produced during these facial exchanges, so this is unlikely to have impacted the results.

Although there was no relationship between facial behaviour and play duration in contrast to previous studies^21,22^, facial mimicry was exact predominantly during gentle play. Perhaps exact facial mimicry helps to signal a readiness to transition into rougher play in sun bears, which is consistent with the proposition that facial communication helps regulate high play intensity^19,22^, and is a pattern previously associated with canid play signalling^19^. However, this possibility requires further research into whether gentle play is more likely to transition into rough play when exact facial mimicry occurs. Alternatively, exact facial mimicry might be more directly linked to gentle play and hereby function, for instance, to strengthen social bonds^24^. Again, this requires further research, involving quantifying social bonds between group members and examining whether exact mimicry is more common among closely bonding dyads.

Altogether, this study provided evidence that sun bears produce facial expressions to communicate in an efficient, effective and exact way. Such complexity in facial communication was previously not known for a non-domesticated, non-primate species and, furthermore, cannot be explained by evolved adaptations to a complex social environment, as these bears are primarily solitary in the wild. Consequently, we suggest the ability to facially communicate in complex ways could be a pervasive trait present across various mammal taxa^25^, allowing mammals to navigate socio-ecologies that can vary in space and time^26^. To explore this possibility, we encourage researchers to test for the presence of this trait in a wide range of mammalian taxa.

### Ethics

Ethics approval was obtained from the University of Portsmouth Animal Research Ethics Committee. BSBCC is a project joined with Land Empowerment Animals People, Sabah Wildlife Department and Sabah Forestry Department. Research at BSBCC is conducted in accordance with their national legal standards of animal care.

## Supplementary information

Video Mimicry

Video Gentle Play

Video Rough Play

Supplementary Methods and Results

## Data Availability

Data is available as a supplementary material.
