# Supplementary material for: Facial Complexity in Sun Bears: Exact Facial Mimicry and Social Sensitivity
Source: Sci Rep. 2019 Mar 21;9:4961. doi: 10.1038/s41598-019-39932-6 (PMC6428817; doi:10.1038/s41598-019-39932-6)
Supplement: Supplementary file 4 — Supplementary Methods and Results [file 41598_2019_39932_MOESM4_ESM.pdf]

Supplementary Information for

Facial Complexity in Sun Bears: Exact Facial Mimicry and Social Sensitivity

D. Taylor, D. Hartmann, G. Dezecache, S. T. Wong, & M. Davila-Ross

Supplementary Methods

Bears and study site

Table S1 provides an overview of the 22 bears (15 females) of this study. The Bornean Sun Bear Conservation Center comprises a total of 2 hectares of natural tropical lowland dipterocarp forest at the edge of the Kabili-Sepilok Forest Reserve, Sabah, Malaysia. Recordings of the bears were collected in three outdoor forest enclosures, ranging from 0.13-0.32 hectares, meaning enclosures were large enough that bears did not have to socially interact by necessity. Group compositions were changed throughout the data collection of this study, but the group sizes within enclosures did not exceed six bears. All bears were fed on a locally sourced herbivorous diet comprising fruits and vegetables such as rambutan, sugar cane, and sweet potato. Keepers fed the animals by scattering the food into the enclosures. Beyond this, staff members seldom intervened in the sun bear activity. To provide as much of a natural ecological setting as possible for the bears, further environmental enrichment was not used. Tourists were often present on high viewing platforms above the enclosures. However, the impact of the presence of tourists on the bears behavior was likely negligible, since they were mostly out of the bears view, and were encouraged to remain quiet so as not to disturb the bears. Water was freely available at all times.

Table S1. Overview of the age and sex of the bears in this study

Sun bear	Age in years	Sex
Ah Bui	6	Female
Ah Lun	7	Female
Boboi	3	Male
Bonkhud	7	Female
Cerah	10	Female
Chin	9	Female
Damai	4	Female
Debbie	5	Female
Fulung	6	Male
Julaini	8	Male
Julita	10	Female
Kala	3	Female
Kitud	3	Female
Koko	4	Female
Kudat	10	Male
Loki	2	Female
Mary	5	Female
Montom	6	Male
Ronnie	12	Male
Sunbearo	2	Male
Susie	10	Female
Tantan	3	Female

Recording Equipment

Two video cameras (Sony HandyCam DCR-TRV19E (Sony Electronics Oradell, NJ, USA) and JVC Everio GZ-MS110BEK (JVC Electronics, Wayne, USA) were used inter-changeably dependent on the individual collecting the recordings and other logistical issues. The recordings were obtained by altogether six recordists.

Behavioural coding

Definitions on open-mouth variants as well as face-to-face, play intensities and play actions are presented in Tables S2-S4, respectively. The coding was conducted by one coder, separately for each bear for the facial expressions, facial orientations and play intensities; play actions were also identified by this coder in order to determine play intensities and play bouts. Coding the behavior of each bear separately was particularly important for the facial expressions; since the coder could not know if and when the facial expressions of the other bear in the play bout was coded, and therefore if facial expressions of play partners were frequently followed by facial expressions in the subject, this could not but attributed to the expectations of the coder. Even if the coder has some expectation that an expression had been coded, it would be very difficult to know precisely at what second, making it unlikely that cases wherein subjects produce an open-mouth expression within 1 second of perceiving an open-mouth expression produced by a play partner were confounded by coder expectations. Inter-coder reliability tests showed Kappa values of .73 for facial variants as well as .80 for facing (both based on 102 expressions) and .75 for play intensity (based on 51 play bouts). Behavioral coding was performed using ELAN (MPI for Psycholinguistics, The Language Archive, Nijmegen, The Netherlands) with a 1-frame accuracy at 25 frames per second.

Table S2. Definitions of open-mouth variants and facing.

Behavior	Definition
NUI expression	Open-mouth expression of play that show no upper incisors (Similar to the jaw-gape face in American Black Bears described by Henry and Herrero, 1974): 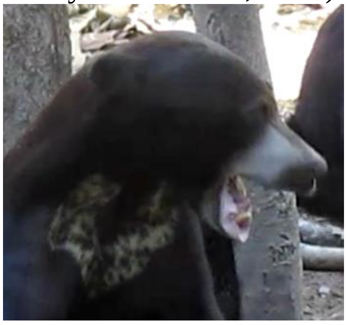
WUI expression	Open-mouth expression of play that show upper incisors. The upper incisors are exposed by raising the nose, which also causes a corresponding wrinkling of the upper muzzle bridge.

Face-to-Face	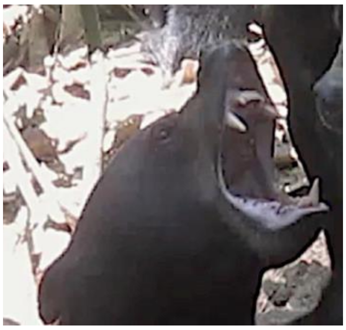<br>Facial orientation when the playing bears were within 45 degree head rotation of one another.

Table S3. Definitions of play intensities.

**Play intensity**	**Definition**
Gentle play	Play with slow sequences of play actions, including shoving, grappling, and leaving-following sequences.
Rough play	Play with fast sequences of play actions, which included all play actions produced during gentle play but faster, in addition to hitting, biting, and flailing.

Table S4. Definitions of play actions, which appeared otherwise functionless. These definitions are partly based on the black bear study by Henry & Herrero (1974).

Play action	Definition		
Shoving	Throwing oneself at a play partner and then withdrawing	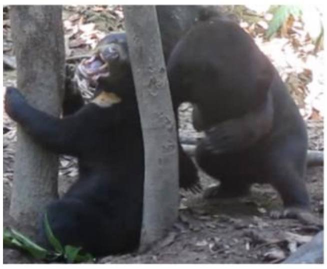	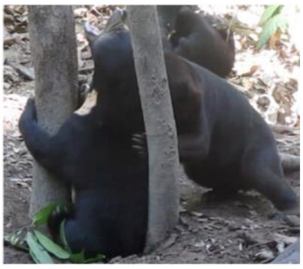
Grappling	Grabbing the play partner, often behind them and/or while biting their nape.	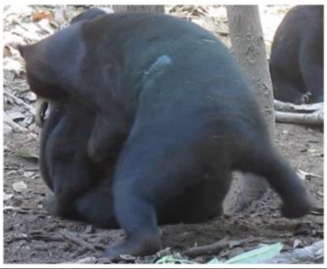	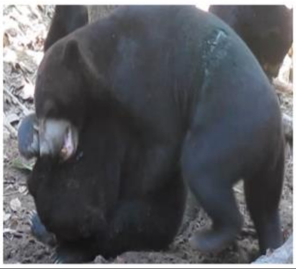

Leaving-following sequences	A behavioral sequence consisting of one play partner leaving and the other following their play partner, often many times consecutively.	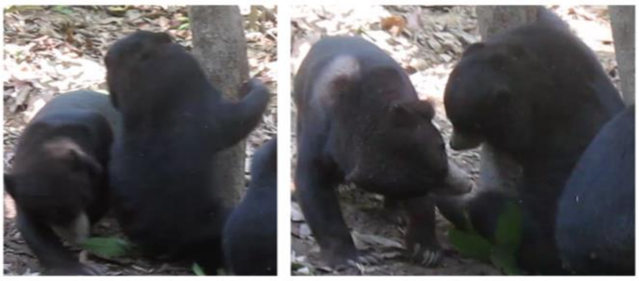
Hitting	Raising the paw and striking the play partner.	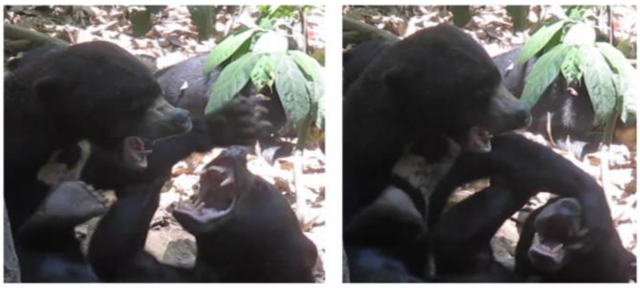
Biting	Biting the play partner, most often on the nape.	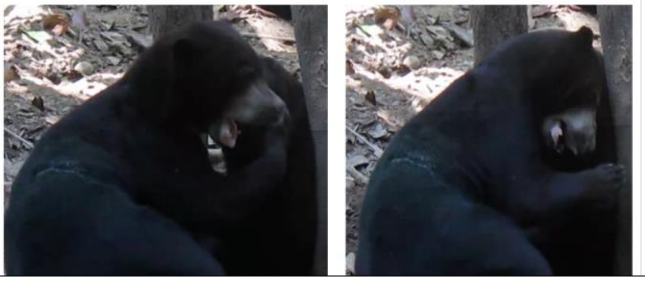
Flailing	Rapidly swinging the head, torso and/or arms from one side to another.	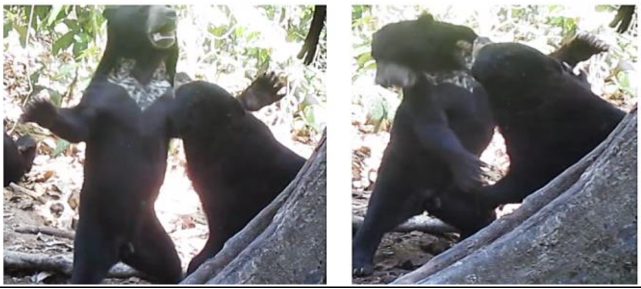

Methods of Supplementary Results

Facial behavior and play duration

Correlations were used to explore whether facial behavior (expression production, mimicry, and exact mimicry) was associated with longer play duration as an indication that this behavior may help to regulate play. Rates of expression production, mimicry and exact mimicry were calculated by dividing the total number of observations of each behavior per subject by the total time spent playing per subject. We then calculated spearman rank correlations between each form of facial behavior and play duration.

NUI and WUI occurrences and play intensity

To ensure that exact facial matching was not an artifact of particular facial variants being produced mostly in one type of play, patterns of expression production Rates of NUI and WUI expression production per subject were calculated by dividing the total number of expressions produced per subject, by the total amount of time spent playing in each play intensity (gentle and rough), which therefore gave 4 rates per subject; the rate of NUI expression production during gentle play, the rate of NUI expression production during rough play, the rate of WUI expression production during gentle play, and the rate of WUI expression production during

rough play. Rates of NUI versus WUI expressions were compared during gentle play, and during rough play, using Mann-Whitney U tests. Rates of NUI variants in gentle versus rough play were compared within-subjects using Wilcoxon Signed-Ranks tests. Rates of WUI variants were also compared within-subjects in gentle versus rough play using Wilcoxon Signed-Ranks tests.

References

1. Henry, J. D., & Herrero, S. M. (1974). Social play in the American black bear: its similarity to canid social play and an examination of its identifying characteristics. *Am. Zool. 14*(1), 371-389.

Supplementary Results

Table S5 shows the percentage of expressions that were mimicked out of all the expressions perceived in a play partner by the subject.

Table S5. Percentage of perceived expressions mimicked per subject.

Subject	Percentage
Ah Bui	37.67
Ah Lun	0
Bonkhud	41.67
Cerah	0
Chin	0
Damai	52.29
Debbie	57.69
Fulung	28.79
Loki	50
Mary	30.4
Montom	50
Panda	100
Sunbearo	70

Table S6 depicts the total number of each case type per subject and shows clearly that several subjects showed a high number of expressions when an expression was perceived, indicating that the observed results do not rest simply on a small number of subjects.

Table S6. Total number of case types per subject.

Subject	Only showing expression in original scene	Only showing expression in matching scene	Always showing expression	Never showing expression
Ah Bui	50	2	5	89
Ah Lun	0	0	0	3
Bonkhud	9	0	1	14
Cerah	0	0	0	1
Chin	0	0	0	1
Damai	55	0	2	52

Debbie	23	4	6	16
Fulung	29	6	9	65
Loki	0	0	0	1
Mary	7	0	0	16
Montom	0	0	0	0
Panda	7	0	0	0
Sunbearo	1	0	0	3

Tables S7 and S8 show the number of WUI and NUI expressions per subject following NUI and WUI expressions of their playmates.

Table S7. Total number of WUI and NUI expressions produced within 1 second of a playmate producing a NUI expression.

Subject	NUI after NUI	WUI after NUI
Ah Bui	21	7
Bonkhud	3	0
Damai	18	2
Debbie	10	8
Fulung	10	8
Mary	0	1
Montom	1	0
Panda	5	1
Sunbearo	4	1
Susie	1	0

Table S8. Total number of WUI and NUI expressions produced within 1 second of a play partner producing a WUI expression.

Subject	WUI after WUI	NUI after WUI
Ah Bui	19	5
Boboi	2	0
Bonkhud	2	1
Damai	27	4
Debbie	20	2
Fulung	10	2
Kitud	0	1
Loki	1	0
Mary	2	0
Montom	2	1
Panda	1	0
Sunbearo	1	3

NUI and WUI occurrences and play intensity

The bears did not produce significantly different rates of the NUI variants versus the WUI variants during gentle play (exact two-tailed Mann-Whitney U, Z= -.572, N= 10 + 9 bears, p = .604) and during rough play (Z= -.082, N=10 + 9, p= .934). Furthermore, neither rates of the NUI variants nor the WUI variants were produced significantly more often in gentle play versus rough play (exact two-tailed Wilcoxon Signed-Ranks; NUI: Z= -.663, T= 21, N= 10, p=.508; WUI: Z= -.178, T = 21, N= 9, p= .859).
